# Physiological Responses to Rifle Carriage During Roller-Skiing in Elite Biathletes

**DOI:** 10.3389/fphys.2019.01519

**Published:** 2019-12-19

**Authors:** Malin Jonsson Kårström, Kerry McGawley, Marko S. Laaksonen

**Affiliations:** The Swedish Winter Sports Research Centre, Mid Sweden University, Östersund, Sweden

**Keywords:** anaerobic energy contribution, biathlon, cross-country skiing, gross efficiency, lactate threshold, oxygen uptake

## Abstract

**Purpose:** This study aimed to investigate the physiological factors affected by rifle carriage during biathlon skiing performance, as well as the sex differences associated with rifle carriage.

**Methods:** Seventeen national- and international-level biathletes (nine females and eight males; age 23.0 ± 3.3 years, $\dot{V}$O_2__max_ 59.4 ± 7.6 mL.kg^–1^.min^–1^) performed a submaximal incremental test and a maximal time-trial (TT) using treadmill roller-skiing (gear 3, skating technique) on two occasions separated by at least 48 h. One condition involved carrying the rifle on the back (WR) and the other condition no rifle (NR) and the tests were randomized. Submaximal $\dot{V}$O_2_, skiing speed at 4 mmol.L^–1^ of blood lactate (speed_@__4__mmol_), gross efficiency (GE), aerobic (MR_ae_), and anaerobic (MR_an_) metabolic rates, and $\dot{V}$O_2__max_ were determined.

**Results:** Submaximal $\dot{V}$O_2_ (at all intensities) and GE (16.7 ± 0.9 vs. 16.5 ± 1.1%) were higher for WR compared to NR (*p* < 0.05), while speed_@__4__mmol_ was lower (3.1 ± 0.4 vs. 3.3 ± 0.5 m.s^–1^, *p* = 0.040). TT performance was improved (4.6 ± 0.4 vs. 4.3 ± 0.4 m.s^–1^, *p* < 0.001) and MR_an_ was higher (31.3 ± 8.0 vs. 27.5 ± 6.5 kJ.min^–1^, *p* < 0.01) for NR compared to WR, with no difference in $\dot{V}$O_2__max_ or MR_ae_. For skiing WR, TT performance was correlated to speed_@__4__mmol_ (*r* = 0.81, *p* < 0.001), MR_an_ (*r* = 0.65, *p* < 0.01), $\dot{V}$O_2__max_ (*r* = 0.51, *p* < 0.05), and relative muscle (*r* = 0.67, *p* < 0.01) and fat (*r* = −0.67, *p* < 0.01) masses. Speed_@__4__mmol_ together with MR_an_ explained more than 80% of the variation in TT performance (WR 84%, NR 81%). Despite a higher relative mass of the rifle in females compared with males (5.6 ± 0.4 vs. 5.0 ± 0.4% of body mass, *p* = 0.012), there were no sex differences associated with rifle carriage measured as absolute or relative differences.

**Conclusion:** Rifle carriage in biathlon skiing led to significantly higher physiological demands during submaximal exercise and reduced performance during maximal treadmill roller-skiing compared to NR for both sexes. The most important variables for performance in biathlon treadmill skiing seem to be speed_@__4__mmol_ combined with MR_an_, both of which were lower for WR compared to NR. To improve skiing performance in biathlon, improving speed at 4 mmol.L^–1^ of blood lactate and anaerobic energy delivery while carrying the rifle are recommended.

## Introduction

Biathlon is an intermittent endurance sport that combines rifle shooting and cross-country (XC) skiing while carrying a rifle. A biathlon competition consists of 6–20 km of XC skiing divided into three or five laps each lasting ∼3–9 min, depending on the competition discipline, and between each lap athletes shoot five shots in a prone or standing position. While few scientific studies have investigated the physiological, biomechanical, technical and tactical demands of biathlon (reviewed in Laaksonen et al., 2018), more have examined XC skiing events (i.e., competing without a rifle). Regarding the physiological demands, biathlon has multiple similarities to XC skiing, especially sprint XC skiing, which also involves repeated bouts of skiing (up to 4 × 2–4 min). However, the rest periods between the bouts are significantly longer in sprint XC races (i.e., 20–45 min) (FIS, 2018). Thus, reported findings on physiological responses in sprint XC skiing studies may not be directly transferrable to skiing performance in biathlon.

As in other endurance sports, maximal oxygen uptake ($\dot{V}$O_2__max_), lactate threshold, efficiency of movement and anaerobic energy delivery all have an important role in biathlon skiing performance (Laaksonen et al., 2018). Indeed, $\dot{V}$O_2__max_ values exceeding 80 mL.*kg*.min^–1^ have been reported for male biathletes, with approximately 15% lower values for females (Tønnessen et al., 2015). Moreover, both $\dot{V}$O_2__max_ and lactate threshold have been shown to correlate with biathlon skiing performance (Rundell, 1995; Rundell and Bacharach, 1995). While gross efficiency (GE) has been shown to correlate well with XC skiing performance (Sandbakk et al., 2013; Andersson et al., 2017), no previous studies have investigated the importance of GE in biathlon when skiing with a rifle. In addition, although performance in biathlon is predominantly dependent on large contributions from aerobic energy delivery, anaerobic energy contributions are also likely to play an important role. This is the case for XC skiing (Stöggl et al., 2007; Andersson et al., 2019), where it has been estimated that ∼ 17–26% of the total energy delivery during maximal XC skiing lasting ∼ 2–4 min is provided by anaerobically-derived energy (Losnegard et al., 2012; McGawley and Holmberg, 2014; Andersson et al., 2016, 2017).

Unlike in XC ski racing, biathletes carry a rifle weighing >3.5 kg on their back while skiing. It has been estimated that the energy cost could be ∼7% higher while carrying an extra load of ∼4 kg while skiing (Fredrick, 1987). Previous studies investigating physiological responses in biathlon have reported a 2–8% higher oxygen uptake ($\dot{V}$O_2_) and a 6–11% higher ventilation rate ($\dot{V}$_E_) among male and female biathletes when carrying a rifle during submaximal roller-skiing compared to no rifle (Rundell and Szmedra, 1998; Stöggl et al., 2015). These differences were also reported to be greater for the female participants compared to the males, which may be due to the higher additional load relative to body mass in females. The two aforementioned previous studies used skiing speeds of between 2.2 and 3.0 m.s^–1^. However, mean skiing speed for the ten best athletes during a biathlon World Cup sprint competition is currently around 6.3 and 7.2 m.s^–1^ for females and males, respectively (including both uphill, downhill, and flat terrain), and this speed has increased by ∼7% over the last 15 years (Laaksonen et al., 2018).

The complex nature of intermittent XC skiing and rifle carriage in biathlon makes the sport unique in its specific demands. With only two studies having investigated the effects of rifle carriage on a limited number of physiological responses in biathlon (Rundell and Szmedra, 1998; Stöggl et al., 2015), more research is needed to fully understand the physiological determinants of biathlon in order to improve sport-specific training and preparation for competition. Therefore, this study aimed to investigate the physiological factors affected by rifle carriage during biathlon skiing performance, as well as the sex differences associated with rifle carriage. This knowledge will help biathletes and coaches to plan and monitor training. It was hypothesized that the physiological responses would be increased when carrying a rifle, and that this effect would be larger in female biathletes compared to males.

## Materials and Methods

### Participants

Seventeen well-trained Swedish biathletes (nine females and eight males), competing at national and international levels, volunteered to participate in the study (Table 1). All participants were fully informed of the nature of the study before providing written consent to participate. The study was pre-approved by the Regional Ethical Review Board in Umeå, Sweden (#2018-122-31M) and was performed in accordance with the Declaration of Helsinki.

**TABLE 1:** Participant characteristics recorded during the seasonal preparation phase (mean ± SD).

	**Total**	**Females**	**Males**	**ES**
	**(*n* = 17)**	**(*n* = 9)**	**(*n* = 8)**	
Age (years)	23.03.3	21.31.4	24.93.9^∗^	0.54
Body height (cm)	1759	1704	1819^∗∗^	0.70
Body mass (kg)	69.76.6	66.34.7	73.66.5^***^	0.61
Muscle mass (kg)	54.98.6	48.63.3	61.96.9^***^	0.84
Muscle mass (% of body mass)	786	734	833^***^	0.79
Upper-body muscle mass (kg)	28.04.0	24.31.5	32.04.0^***^	0.84
Upper-body muscle mass (% of total muscle mass)	402	372	442^∗^	0.58
Fat mass (kg)	12.54.0	15.43.1	9.31.8^***^	0.77
Fat mass (% of body mass)	186	234	133^***^	0.79
Upper-body length (cm)	724	715	733	0.26
Lower-body length (cm)	1038	996	1087^∗^	0.56
Training load (hours.year^–1^)	647133	623153	69082	0.30
Participation in biathlon competitions (years)	114	103	126	0.16
Training with rifle (hours.week^–1^)	3.92.5	4.11.7	3.63.8	0.29
Training with rifle (% of total training time)	3018	3314	2526	0.41

*Different to females: ^∗^*p* < 0.05, ^∗∗^*p* < 0.01, ^∗∗∗^*p* < 0.001. ES, effect size.*

### Study Overview

Participants visited the laboratory for testing on two separate occasions during the preparation phase of the season (October), with 2–6 days (and a minimum of 48 h) separating the visits. The two testing sessions were performed in a randomized order and involved the same submaximal and maximal exercise protocol, one test skate roller-skiing while carrying the rifle on the back (WR) and one test without the rifle (NR). All participants used the same rifle during testing, which weighed 3.5 kg, and their own biathlon harness weighing 0.2 kg. The testing was performed on a treadmill using gear 3, which all participants were highly familiar with from previous testing and training sessions.

### Testing Procedures

The skiing tests were carried out on a motor-driven treadmill (belt dimensions 3.3 × 2.5 m; Rodby Innovation AB, Vänge, Sweden). Participants wore a safety harness around their waist, which was suspended from the ceiling and connected to an emergency brake stopping the treadmill within 1 s in the case of a fall. All participants used Pro-Ski S2 roller skis (Sterners, Dala-Järna, Sweden) equipped with NNN (Rottefella, Klockarstua, Norway, rolling resistance 0.0224) or SNS (Salomon, Annecy, France, rolling resistance 0.0216) bindings. The roller-skis were pre-warmed for at least 60 min in a heating box before all tests to minimize the variation in rolling resistance (μ_R_), which was determined as described previously by Ainegren et al. (2008). Expired air was sampled at 10-s intervals during submaximal and maximal exercise using an AMIS 2001 metabolic system (model C, Innovision A/S, Odense, Denmark), which was calibrated before each testing session using a 3-L syringe and a calibration gas with a known mixture of 16% O_2_ and 4.5% CO_2_.

On arrival at the laboratory participants rested in a supine position for 10 min before a capillary blood sample was collected from the fingertip for the subsequent analysis of resting blood lactate concentration (Biosen 5140, EKF Diagnostic GmbH, Magdeburg, Germany). Body mass and height were also measured (Seca 764, Hamburg, Germany). Thereafter, participants performed a standardized warm-up by roller-skiing for 6 min at the same workload as for the first submaximal exercise level during the subsequent test. The submaximal test consisted of 3–5 levels each lasting 4 min where the gradient was constant (3.5° for the females, 4.5° for males) and the speed increased by 0.56 m.s^–1^ (2 km.h^–1^) for every workload. The female skiers commenced at a velocity of 1.94 m.s^–1^ (7 km.h^–1^), while the males commenced at 2.22 m.s^–1^ (8 km.h^–1^). Expired air was analyzed from measurements taken during the last 30 s of each 4-min level and heart rate (HR) was recorded throughout the submaximal test (Polar S810, Polar Electro Oy, Kempele, Finland). Between each level, the treadmill was stopped for 60 s for fingertip blood sampling and to record the rating of perceived exertion (RPE; Borg-scale 6-20) for breathing, arms (RPE_arm_) and legs (RPE_leg_). The submaximal test was terminated when the respiratory exchange ratio (RER) was >1.00, the $\dot{V}$_E__/_$\dot{V}$O_2_ ratio was >30 and HR was >90% of the known maximal HR reported by the participant.

After the submaximal test participants recovered actively for 4 min (roller-skiing at the first submaximal workload) then rested passively for 5 min. Participants then completed 5 min of treadmill roller-skiing as a re-warm-up, including three sprints lasting 15 s, before commencing a maximal time trial (TT). The TT simulated competition skiing conditions, whereby the females completed 900 m at a gradient of 3.5° and the males completed 1000 m at 4.5° as fast as possible. The participants were able to adjust the speed by moving forward and backward on the treadmill, with a system consisting of two laser beams detecting the position of the participant (Swarén et al., 2013). The speed of the treadmill increased by 0.19 m.s^–1^.s^–1^ or decreased by 0.11 m.s^–1^.s^–1^ as the participant moved to the front or rear of the treadmill, respectively. During the TT, expired air and HR were measured throughout and a fingertip blood sample was collected 2 min after the test for the analysis of maximal blood lactate concentration. Performance was defined as mean speed during the TT.

Within 1–3 days of the roller-skiing tests body composition was assessed using iDXA (Encore 2007, Version 11.4, General Electric Company, Madison, WI, United States) in order to measure total body mass, as well as relative and absolute muscle and fat masses. The participants were tested in a fasted state (in the morning before breakfast) and lay in a supine position during the scan, which lasted 7–8 min. When analyzing the iDXA data, the body was also divided into upper- and lower-body sections. Arms, legs, and trunk were also analyzed separately for body composition and the length of the different body segments were measured. The length of the upper-body was measured without the head.

### Calculations

Respiratory values ($\dot{V}$O_2_, $\dot{V}$CO_2_, $\dot{V}$_E_, and RER) and HR were calculated as the mean of the last 30 s of each workload during the submaximal test. Speed, HR and $\dot{V}$O_2_ corresponding to a blood lactate concentration of 4 mmol.L^–1^ (speed_@__4__mmol_, HR_@__4__mmol_, and $\dot{V}$O_2__@__4__mmol_, respectively) were calculated from the individual exponential relationships between blood lactate concentration and speed, $\dot{V}$O_2_ and HR. Heart rate, $\dot{V}$O_2_, $\dot{V}$CO_2_, and $\dot{V}$_E_ were recorded throughout the TT and the highest consecutive 30-s values were reported as maximal values. Power output (PO) during roller-skiing was calculated as the power exerted to elevate the total mass against gravity and to overcome the rolling resistance:

(1) PO(W)=M×totg×v(sin(α)+μ×Rcos(α))

where M_tot_ = body mass (kg) including equipment (also the rifle, if used), g = gravitational acceleration (m.s^–2^), v = velocity (m.s^–1^), μ_R_ = rolling resistance and α = treadmill incline (°) (Andersson et al., 2016).

The aerobic metabolic rate (MR_ae_) was determined from the mean $\dot{V}$O_2_ and RER during the last minute of the highest submaximal workload with a RER < 1.00:

(2) MR(W)ae=(E×GROSS4184)/60

where E_GROSS_ = gross energy expenditure, given by:

(3) E(kcal.min)-1GROSS=(1.1×RER+3.9)×V.O(L.min)-12

Gross efficiency (GE) was calculated from the submaximal workload closest to, but not above, RER = 1.00:

(4) GE(%)=(PO/MR)ae×100

The total metabolic demand (MR_DEMAND_) of the TT was calculated using the GE method:

(5) MR(W)DEMAND=PO(W)TT/GE

where PO_TT_ was calculated using Eq. 1 with the mean speed for the TT. The MR_ae_ for the TT was calculated using Eqs. 2 and 3 and the mean $\dot{V}$O_2_ for the entire TT, assuming RER = 1.0. Anaerobic metabolic rate (MR_an_) was then calculated as follows:

(6) MR(W)an=MR-DEMANDMRae

E_GROSS_ was calculated for the first three submaximal levels using values for $\dot{V}$O_2_ (L.min^–1^) and RER values ≤ 1.00 using Eq. 3.

### Statistical Analyses

All statistical tests were processed using SPSS 24.0 Software (SPSS, Inc., Chicago, IL, United States). Shapiro-Wilk tests showed a normal distribution for all variables for the group in total. For analyses separated for sex, non-parametric tests were used due to the small sample sizes. For the submaximal test, a two-way ANOVA with repeated measures (skiing condition [WR/NR] × submaximal level) was used to investigate differences in respiratory variables, HR and energy system contributions. If a significant difference was observed, repeated measures one-way ANOVA with a Bonferroni *post hoc* test was used to identify specific differences for the group in total, while a Friedman’s test followed by a *post hoc* Wilcoxon signed rank test was used on RPE and when the sexes were tested separately. Paired samples *T*-tests (for the group in total) and Wilcoxon Signed Rank tests (for females and males separately) were used to determine the differences between WR and NR for respiratory variables ($\dot{V}$O_2_, RER, and $\dot{V}$_E_), HR and blood lactate during the maximal TT. Responses to rifle carriage were calculated as the absolute and relative differences between WR and NR and were compared between the sexes using a Mann–Whitney *U* test. Effect sizes (ES) were calculated using Cohen’s *d* with the criteria 0.2 = small effect, 0.5 = moderate effect, and 0.8 = large effect. To analyze the relationship between TT performance (dependent variable) and the independent variables for endurance performance (speed_@__4__mmol_, $\dot{V}$O_2__max_, GE, MR_an_, MR_ae_, muscle mass, and fat mass), Pearson’s product moment correlation tests were conducted for WR. When separating the group by sex, Spearman’s Rho was used due to the small sample size. In addition, a multiple regression analysis was conducted with speed_@__4__mmol_, $\dot{V}$O_2__max_, GE, MR_an_ and muscle mass as independent variables to determine the explained variation in TT performance. The TT was also divided into four quarters according to distance covered (i.e., 225 and 250 m for the females and males, respectively) in order to analyze the relationship between aerobic and anaerobic energy contributions during different sections of the TT. Mean PO_TT_, speed, MR_ae_ and MR_an_ were compared using a two-way repeated measures ANOVA (skiing condition [WR/NR] × quarter), followed by a one-way repeated measures ANOVA with a Bonferroni *post hoc* test (for the group in total) or a Friedman’s test followed by a *post hoc* Wilcoxon signed rank test (for the females and males separately). The level of statistical significance was set at α ≤ 0.05 and data are presented as mean ± standard deviation (SD), except for RPE, where data are presented as median and interquartile range (IQR).

## Results

### Submaximal Test

Responses during submaximal exercise for WR and NR are presented in Table 2. All participants (*n* = 17) performed the first three levels of the submaximal test for both WR and NR, while only 14 participants (eight females, six males) performed four submaximal levels for NR and 12 of those (seven females, five males) performed four levels for WR.

**TABLE 2:** Responses to skiing with (WR) and without (NR) the rifle during four submaximal levels (Sub 1–4).

	**Sub 1 (*n* = 17)**		**Sub 2 (*n* = 17)**		**Sub 3 (*n* = 17)**		**Sub 4 (WR *n* = 12, NR *n* = 14)**	
**All participants**	**WR**	**NR**	**WR**	**NR**	**WR**	**NR**	**WR**	**NR**
$\dot{V}$O_2_ (L.min^–1^)	2.60.5^2−4^	2.50.5^2−4,*^	3.10.6^1,3,4^	3.00.6^1,3,4,*^	3.60.7^1,2,4^	3.50.7^1,2,4,*^	4.00.8^1−3^	3.80.7^1−3,*^
$\dot{V}$CO_2_ (L.min^–1^)	2.40.6^2−4^	2.30.5^2−4,*^	2.90.6^1,3,4^	2.80.7^1,3,4,*^	3.60.8^1,2,4^	3.50.7^1,2,4,*^	4.20.9^1−3^	4.00.8^1−3,*^
$\dot{V}$_E_ (L.min^–1^)	72.317.0^2−4^	67.116.4^2−4,*^	88.719.9^1,3,4^	82.520.1^1,3,4,*^	112.526.7^1,2,4^	105.224.6^1,2,4,*^	133.531.3^1−3^	122.224.3^1−3,*^
HR (beats.min^–1^)	14314^2−4^	14315^2−4^	16016^1,3,4^	15915^1,3,4^	17414^1,2,4^	17313^1,2,4^	17911^1−3^	17910^1−3^
RER	0.910.05^2−4^	0.900.05^2−4^	0.940.06^1,3,4^	0.930.05^1,3,4^	1.000.07^1,2,4^	0.990.07^1,2,4^	1.050.04^1−3^	1.040.05^1−3^
Blood lactate (mmol.L^–1^)	1.61.1^2−4^	1.71.0^2−4^	2.41.7^1,3,4^	2.41.6^1,3,4^	4.62.6^1,2,4^	4.12.2^1,2,4^	7.01.2^1−3^	6.11.4^1−3,*^
E_GROSS_ (kcal.min^–1^)	12.72.7^2,3^	12.22.5^2,3,*^	15.42.9^1,3^	14.73.0^1,3,*^	17.93.3^1,2^	17.33.3^1,2,*^	–	–

**Females**	**Sub 1 (*n* = 9)**		**Sub 2 (*n* = 9)**		**Sub 3 (*n* = 9)**		**Sub 4 (WR *n* = 7, NR *n* = 8)**	

$\dot{V}$O_2_ (L.min^–1^)	2.20.2^2−4^	2.10.2^2−4,*^	2.70.2^1,3,4^	2.50.2^1,3,4,*^	3.10.2^1,2,4^	3.00.2^1,2,4,*^	3.40.2^1−3^	3.30.2^1−3,*^
$\dot{V}$CO_2_ (L.min^–1^)	1.90.2^2−4^	1.80.1^2−4,*^	2.40.2^1,3,4^	2.30.2^1,3,4,*^	3.00.3^1,2,4^	2.90.3^1,2,4,*^	3.60.2^1−3^	3.50.3^1−3,*^
$\dot{V}$_E_ (L.min^–1^)	59.45.1^2−4^	55.15.9^2−4,*^	72.86.3^1,3,4^	67.68.4^1,3,4,*^	92.711.8^1,2,4^	87.711.1^1,2,4^	111.711.9^1−3^	107.014.4^1−3^
HR (beats.min^–1^)	14213^2−4^	14313^2−4^	15913^1,3,4^	16013^1,3,4^	17312^1,2,4^	17411^1,2,4^	18112^1−3^	1829^1−3^
RER	0.890.05^2−4^	0.880.03^2−4^	0.920.04^1,3,4^	0.900.03^1,3,4^	0.980.05^1,2,4^	0.980.04^1,2,4^	1.050.05^1−3^	1.040.04^1−3^
Blood lactate (mmol.L^–1^)	1.20.4^2−4^	1.30.3^2−4^	1.80.3^1,3,4^	1.80.4^1,3,4^	3.70.9^1,2,4^	3.51.0^1,2,4^	7.11.5^1−3^	6.31.8^1−3^
E_GROSS_ (kcal.min^–1^)	10.60.8^2,3^	10.20.8^2,3,*^	13.00.9^1,3^	12.41.0^1,3,*^	15.31.0^1,2^	14.81.0^1,2,*^	–	–

**Males**	**Sub 1 (*n* = 8)**		**Sub 2 (*n* = 8)**		**Sub 3 (*n* = 8)**		**Sub 4 (WR *n* = 5, NR *n* = 6)**	

$\dot{V}$O_2_ (L.min^–1^)	3.10.4^2−4^	3.00.3^2−4,*^	3.70.3^1,3,4^	3.50.4^1,3,4,*^	4.20.5^1,2,4^	4.10.5^1,2,4,*^	4.80.6^1−3^	4.50.5^1−3,*^
$\dot{V}$CO_2_ (L.min^–1^)	2.80.4^2−4^	2.70.4^2−4^	3.50.4^1,3,4^	3.30.6^1,3,4,*^	4.30.6^1,2,4^	4.10.6^1,2,4,*^	5.00.7^1−3^	4.70.6^1−3,*^
$\dot{V}$_E_ (L.min^–1^)	86.813.5^2−4^	80.613.5^2−4,*^	106.612.9^1,3,4^	99.315.2^1,3,4,*^	134.919.7^1,2,4^	125.020.1^1,2,4,*^	164.022.0^1−3^	142.519.4^1−3,*^
HR (beats.min^–1^)	14417^2−4^	14217^2−4^	16019^1,3,4^	15819^1,3,4^	17516^1,2,4^	17316^1,2,4^	1779^1−3^	17913^1−3^
RER	0.920.06^3,4^	0.930.05^3,4^	0.960.07^4^	0.950.07^3,4^	1.020.09^1,4^	1.010.09^1,2,4^	1.050.03^1−3^	1.030.05^1−3^
Blood lactate (mmol.L^–1^)	1.91.5^2−4^	2.21.4^2−4^	3.12.4^1,3,4^	3.12.1^1,3,4^	5.63.5^1,2,4^	4.93.0^1,2,4,*^	6.80.8^1−3^	5.81.0^1−3,*^
E_GROSS_ (kcal.min^–1^)	15.21.8^2,3^	14.51.7^2,3,*^	18.11.8^1,3^	17.32.2^1,3,*^	20.92.3^1,2^	20.22.3^1,2,*^	–	–

*Data are presented as mean ± SD. Different from submaximal level 1^1^, 2^2^, 3^3^, and 4^4^ for the same condition (WR/NR), *P* < 0.05. Different from WR for the same submaximal level: ^∗^*P* < 0.05. $\dot{V}$O_2_, oxygen uptake; $\dot{V}$_*E*_, ventilation rate; HR, heart rate; RER, respiratory exchange ratio; E_*GROSS*_, gross energy expenditure.*

**FIGURE 1:**
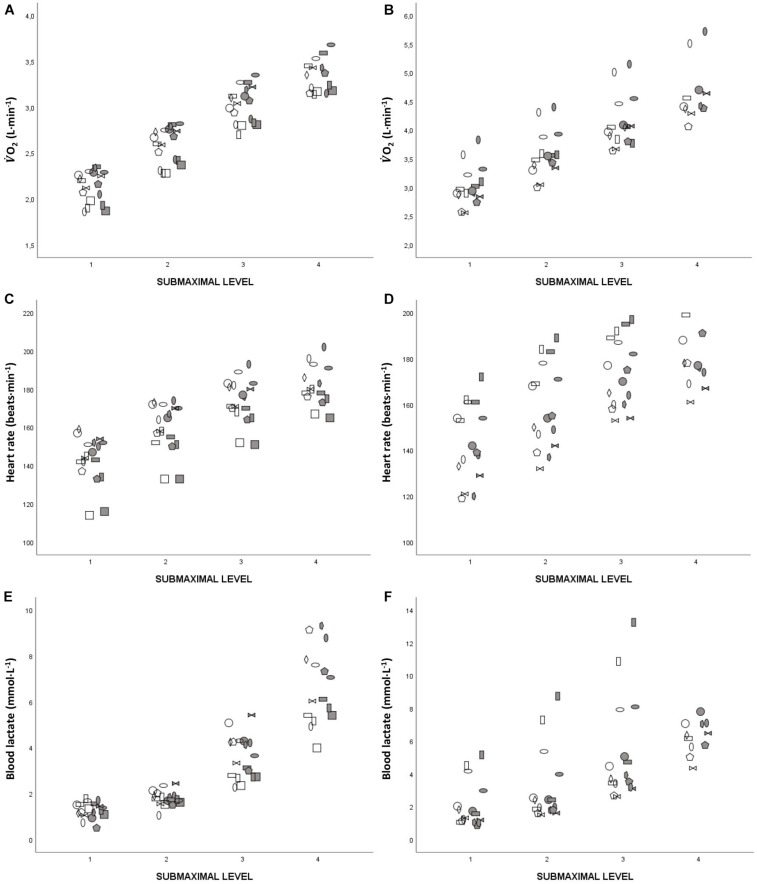
**(A–F)** Individual responses during the first four submaximal levels for $\dot{V}$O_2_ (**A**, females; **B**, males), heart rate (**C**, females; **D**, males), and blood lactate (**E**, females; **F**, males). Filled markers represent skiing with the rifle (WR) and unfilled markers represent skiing without the rifle (NR).

The $\dot{V}$O_2_ was 3–5% higher for WR than for NR during submaximal levels 1–4. The $\dot{V}$CO_2_ was 4–6% higher and $\dot{V}$_E_ was 7–10% higher, while there were no differences in HR or RER. The blood lactate concentration was higher for WR compared to NR only for the fourth submaximal level. The only difference in RPE between WR and NR was for submaximal levels 2 (RPE_arm_ 13 (12–15) vs. 12 (11–13), *p* < 0.05) and 3 (RPE_leg_ 15 (14–15) vs. 14 (12–15), *p* < 0.05). The individual values for submaximal $\dot{V}$O_2_, HR and blood lactate for WR and NR are shown in Figures 1A–F. For the group in total (both sexes), E_GROSS_ was higher during WR compared to NR for all submaximal levels. GE was also higher during WR compared to NR for the group in total (16.7 ± 0.9 vs. 16.5 ± 1.1%, *p* < 0.05, ES = 0.54). No interaction effects were found between skiing condition (WR vs. NR) and submaximal level for any of the variables.

The absolute and relative values for $\dot{V}$O_2__@__4__mmol_ and HR_@__4__mmol_ were similar between WR and NR for the group in total (absolute $\dot{V}$O_2__@__4__mmol_ 3.5 ± 0.7 vs. 3.4 ± 0.7 L.min^–1^, ES = 0.32; relative $\dot{V}$O_2__@__4__mmol_ 84 ± 4 vs. 83 ± 5% of $\dot{V}$O_2__max_, ES = 0.41; absolute HR_@__4__mmol_ 171 ± 10 vs. 173 ± 10 beats.min^–1^, ES = 0.21; relative HR_@__4__mmol_ 91 ± 3 vs. 91 ± 4% of HR_max_, ES = 0.07; all *p* > 0.05). However, speed_@__4__mmol_ was higher during NR compared with WR (3.3 ± 0.5 vs. 3.1 ± 0.4 m.s^–1^, *p* = 0.040, ES = 0.35).

While the mass of the rifle in relation to body mass was greater for the females than the males (5.6 ± 0.4 vs. 5.0 ± 0.4% of body mass, respectively; *p* = 0.012), there were no sex differences in the responses to rifle carriage (i.e., absolute or relative differences between WR and NR) for HR, $\dot{V}$O_2_, $\dot{V}$_E_, RER, $\dot{V}$O_2__@__4__mmol_, HR_@__4__mmol_, speed_@__4__mmol_, or blood lactate during the submaximal test. The males had a higher absolute $\dot{V}$O_2__@__4__mmol_ than the females for both WR and NR (both *p* = 0.002), and a higher GE during NR (17.0 ± 1.3% for males vs. 16.0 ± 0.5% for females, *p* < 0.05), but there was no sex difference in GE for WR.

### Maximal TT

**FIGURE 2:**
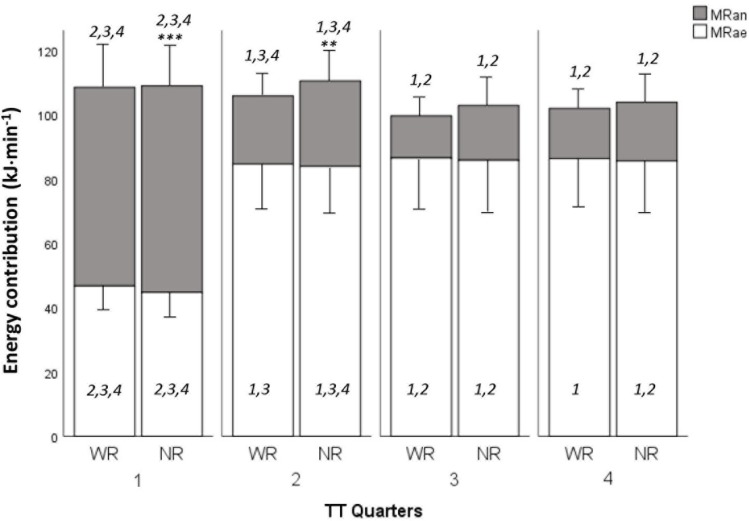
Aerobic (MR_ae_) and anaerobic (MR_an_) energy contributions for each quarter (1–4) of the maximal time trial (TT: 225 m each for females, 250 m each for males) when skiing with (WR) and without (NR) the rifle. Different from NR: ^∗∗^*p* < 0.01, ^∗∗∗^*p* < 0.001. Numbers indicate a difference from other quarters for the same condition (WR/NR), *P* < 0.05.

**FIGURE 3:**
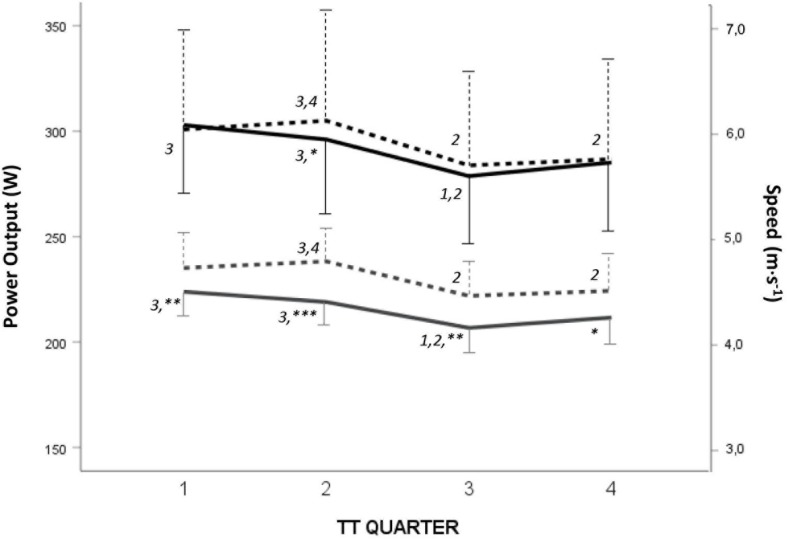
Mean power output (black lines) and speed (gray lines) for each quarter (1–4) of the maximal time trial (TT: 225 m each for females, 250 m each for males) when skiing with (WR; intact line) and without (NR; dashed line) the rifle. Different from NR: ^∗^*p* < 0.05, ^∗∗^*p* < 0.01, ^∗∗∗^*p* < 0.001. Numbers indicate a difference from other quarters for the same condition (WR/NR), *P* < 0.05.

Responses during the maximal TT for WR and NR are presented in Table 3. Time to complete the TT was 14.3 ± 9.4 s longer for WR compared to NR (*p* < 0.001; *n* = 17). No differences were found in $\dot{V}$O_2__max_ between WR and NR, whereas MR_an_ (both absolute and relative) was higher for NR (Table 3). No differences in maximal RPE (for breathing, arms, or legs) were observed between WR and NR. Compared to the female athletes, the males had a higher $\dot{V}$O_2__max_ (absolute and relative),$\dot{V}$_E_ and absolute MR_ae_ during the TT for both WR and NR, and a higher absolute MR_an_ for WR (Table 3). The differences in mean speed and mean power output between the four quarters and the two skiing conditions (WR/NR) during the TT for the group in total are illustrated in Figure 2. There was no difference in MR_ae_ between WR and NR during the TT, while the absolute MR_an_ was higher during the first half of the TT for NR (Figure 3).

**TABLE 3:** Responses to skiing with (WR) and without (NR) the rifle during the maximal time trial.

**All participants (*n* = 17)**	**WR**	**NR**	**ES**
Time (s)	221.621.6	207.320.3^***^	1.52
Mean speed (m.s^–1^)	4.30.4	4.60.4^***^	1.55
$\dot{V}$O_2__max_ (L.min^–1^)	4.20.8	4.20.8	0.07
$\dot{V}$O_2__max_ (mL.kg^–1^.min^–1^)	56.16.7	59.47.6^***^	1.20
VE_max_ (L.min^–1^)	159.635.8	161.137.4	0.28
HR_max_ (beats.min^–1^)	18910	1909	0.39
Maximal blood lactate (mmol.L^–1^)	13.22.9	14.02.6	0.32
MR_an_ (kJ.min^–1^)	27.56.5	31.38.0^∗∗^	0.77
MR_an_ (%)	263	295^∗∗^	0.80
MR_ae_ (kJ.min^–1^)	76.614.0	75.014.2	0.39
MR_ae_ (%)	743	715^∗∗^	0.80
**Females (*n* = 9)**			
Time (s)	216.117.9	202.415.7^∗∗^	0.63
Mean speed (m.s^–1^)	4.20.3	4.50.3^∗∗^	0.63
$\dot{V}$O_2__max_ (L.min^–1^)	3.60.2	3.60.2	0.03
$\dot{V}$O_2__max_ (mL.kg^–1^.min^–1^)	50.73.1	53.44.0^∗^	0.52
VE_max_ (L.min^–1^)	130.412.9	131.210.2	0.17
HR_max_ (beats.min^–1^)	1889	1898	0.30
Maximal blood lactate (mmol.L^–1^)	12.22.0	13.02.8	0.13
MR_an_ (kJ.min^–1^)	23.64.0	27.86.4^∗^	0.52
MR_an_ (%)	273	305^∗^	0.52
MR_ae_ (kJ.min^–1^)	65.13.2	63.63.8	0.27
MR_ae_ (%)	733	705^∗^	0.52
**Males (*n* = 8)**			
Time (s)	227.824.5	212.724.4^∗^	0.60
Mean speed (m.s^–1^)	4.40.4	4.80.5^∗^	0.60
$\dot{V}$O_2__max_ (L.min^–1^)	4.90.5^‡⁣‡‡^	4.90.6^‡⁣‡‡^	0.07
$\dot{V}$O_2__max_ (mL.kg^–1^.min^–1^)	62.13.8^‡⁣‡‡^	66.04.4^*,‡⁣‡‡^	0.63
VE_max_ (L.min^–1^)	192.420.5^‡⁣‡‡^	194.825.2^‡⁣‡‡^	0.23
HR_max_ (beats.min^–1^)	19110	19210	0.16
Maximal blood lactate (mmol.L^–1^)	14.43.5	15.12.0	0.07
MR_an_ (kJ.min^–1^)	31.86.2^‡‡^	35.38.2	0.32
MR_an_ (%)	263	283	0.40
MR_ae_ (kJ.min^–1^)	89.59.0^‡⁣‡‡^	87.89.3^‡⁣‡‡^	0.21
MR_ae_ (%)	743	723	0.40

*Data are presented as mean ± SD. Different from WR: ^∗^*p* < 0.05, ^∗∗^*p* < 0.01, ^∗∗∗^*p* < 0.001. Different from females for the same condition (WR/NR): ^‡⁣‡^*p* < 0.001, ^‡⁣‡⁣‡^*p* < 0.001. $\dot{V}$O_2_, oxygen uptake; $\dot{V}$_*E*_, ventilation rate; HR, heart rate; MR_*an*_, Anaerobic metabolic rate; MR_*ae*_, Aerobic metabolic rate; ES, effect size.*

### Relationships to Maximal TT Performance

When skiing WR, bivariate correlation analyses showed that speed_@__4__mmol_ (*r* = 0.81, *p* < 0.001), relative $\dot{V}$O_2__max_ (*r* = 0.69, *p* < 0.01), MR_an_ (*r* = 0.65, *p* < 0.01), and relative muscle (*r* = 0.67, *p* < 0.01) and fat (*r* = −0.67, *p* < 0.01) masses were significantly correlated with TT performance for the group in total. When sexes were separated, speed_@__4__mmol_ (*r* = 0.95, *p* < 0.001), relative $\dot{V}$O_2__max_ (*r* = 0.83, *p* < 0.01), MR_an_ (*r* = 0.72, *p* < 0.05) and relative muscle (*r* = 0.87, *p* < 0.01) and fat (*r* = −0.87, *p* < 0.01) masses were associated with TT performance for the females, while only relative $\dot{V}$O_2__max_ (*r* = 0.91, *p* < 0.01) was correlated for the males. The multiple regression analyses showed that for WR, speed_@__4__mmol_ and MR_an_ together explained 84% (66% and 18%, respectively; *p* = 0.003) of the variation in performance. When adding absolute muscle mass, GE and $\dot{V}$O_2__max_ to this model, 91% of the variation in performance was explained (all *p* > 0.05). The multiple regression model for NR presented similar results, with speed_@__4__mmol_ and MR_an_ explaining 81% (43% and 38%, respectively; *p* = 0.004) of the variation in performance during the TT, and when adding absolute muscle mass, GE and $\dot{V}$O_2__max_ the predictive value of the model increased to 88% (all *p* > 0.05).

## Discussion

This study aimed to investigate the physiological factors affected by rifle carriage during biathlon skiing performance, as well as the sex differences associated with rifle carriage. The main findings showed that: (1) rifle carriage affects respiratory responses, GE and speed_@__4__mmol_ during submaximal treadmill roller-skiing, as well as the anaerobic energy contribution and performance during a maximal roller-skiing TT; (2) speed_@__4__mmol_ and anaerobic energy contribution seem to explain the majority of the variation in maximal TT performance lasting ∼ 3–4 min; and (3) although the mass of the rifle was greater relative to body mass for the female athletes, there were no sex differences in the physiological responses to rifle carriage.

Previous studies investigating the physiological response to rifle carriage have reported average treadmill roller-skiing speeds of 2.22–2.97 m.s^–1^ (Rundell and Szmedra, 1998; Stöggl et al., 2015), while average speeds in the present study were 1.94–4.19 m.s^–1^ for females and 2.22–4.43 m.s^–1^ for males during the submaximal and maximal tests. The difference in top average speeds reported in the present study compared to the study by Stöggl et al. (2015), both of which involved skiing at competition speeds, can be at least partly explained by the steeper treadmill incline used in the study by Stöggl et al. (2015), which was 5° compared to 3.5–4.5° in the present study. While the average TT speeds in the present study were more closely matched to real-world competition speeds than those reported in previous studies, they are still much lower than the mean speeds of 6.3 and 7.2 m.s^–1^ for females and males, respectively, reported by Laaksonen et al. (2018) for World Cup competitions. However, it is important to recognize that these speeds were derived from the top ten athletes in the World Cup, and that World Cup races include all types of terrain (i.e., flat, uphill, and downhill).

During submaximal exercise $\dot{V}$O_2_, $\dot{V}$CO_2_, $\dot{V}$_E_, and E_GROSS_ were higher for WR compared to NR, which is consistent with findings reported by Stöggl et al. (2015) and Rundell and Szmedra (1998). In addition, blood lactate concentration was higher for WR compared to NR following the fourth submaximal level for the group in total as well as for the males, suggesting that the aerobic energy system is able to meet the energy demands at lower intensities for WR, while this is not possible at higher workloads. This is consistent with previous studies, where differences in blood lactate concentrations were demonstrated for workloads around or above the lactate threshold (Rundell and Szmedra, 1998; Stöggl et al., 2015). However, it is relevant to note that the inter-individual differences in blood lactate responses when skiing WR and NR were large (see Figure 1) and it may therefore be beneficial to individualize training while carrying the rifle depending on the blood lactate responses of each athlete.

Biathletes and XC skiers often use HR monitoring to control exercise intensities when training. In the present study, no differences in HR were observed between WR and NR, although the respiratory responses $(\dot{V}$O_2_, $\dot{V}$CO_2_, $\dot{V}$_E_) were higher during WR. Day-to-day variations in HR of up to 4.1% have been reported during submaximal exercise (Taylor, 1944). Therefore, both HR and blood lactate concentrations are likely not sensitive enough to detect the differences in respiratory responses between WR and NR. This is important to consider when prescribing and analyzing training loads, since the higher metabolic demands of skiing WR will probably not be reflected by HR recordings or sampling of blood lactate concentrations following a training or racing bout. Thus, more accurate methods of monitoring and quantifying training intensities in biathlon, particularly when skiing WR, are required. Methods for calculating power output are currently being investigated within XC skiing, which could improve training-load quantification. Such possibilities need further scientific investigation within biathlon.

Previous research has shown that around 60% of performance in biathlon sprint competitions can be explained by skiing speed (Luchsinger et al., 2018; Dzhilkibaeva et al., 2019), with the remaining 40% explained by the shooting time and accuracy. Speed at the lactate threshold, $\dot{V}$O_2__max_, GE, and anaerobic energy contribution are suggested to be the most important factors for performance in endurance-based sports (Joyner and Coyle, 2008), including XC and biathlon skiing (Rundell, 1995; Rundell and Bacharach, 1995; Larsson et al., 2002; Carlsson et al., 2012, 2016). Therefore, these variables together with muscle mass were used as independent variables in the multiple regression analyses in the present study. Results showed that the most important factors for skiing performance in a TT lasting ∼ 3–4 min were the speed at 4 mmol⋅L^–1^ of blood lactate and the anaerobic energy contribution, which is supported by previous research (Rundell, 1995; Rundell and Bacharach, 1995; Andersson et al., 2017).

The reported training load performed with the rifle was approximately 4 h.week^–1^ (i.e., ∼ 30% of the endurance-based training) for the participants in the present study, but the individual variation was large (ranging from 1–10 h.week^–1^). The speed_@__4__mmol_ and the anaerobic energy contributions were lower during WR compared to NR, which may be a result of the lower training load performed while carrying the rifle on the back. Although not investigated in the present study, anecdotal reports indicate that biathletes often perform high-intensity training without carrying the rifle, which may therefore affect developments in anaerobic energy contribution. These findings suggest that training to improve speed_@__4__mmol_ and anaerobic energy contribution while skiing with the rifle may help to improve skiing performance while carrying the rifle. While speculative, it may be hypothesized that increasing the relative training load with the rifle may be required by biathletes in order to improve biathlon skiing performance.

The duration of the TT in the present study corresponds to the ski-lap durations in a biathlon single mixed-relay (∼ 3–3.5 min per lap). Although the skiing laps are longer in other biathlon disciplines, the importance of the speed_@__4__mmol_ and anaerobic energy contribution for performance may still play an important role due to the variation in terrain and the requirement to increase speed rapidly (e.g., sprinting to the finish line). Given the intermittent nature of biathlon skiing, the variation in terrain (which changes continuously between uphill, downhill, and flat sections) and the variation in competition duration and formats, more research is needed to understand the specific demands and energy system contributions during biathlon competitions, particularly with respect to the unique requirement for rifle carriage (compared to XC skiing, for example).

In biathlon races, the sex difference in skiing speed is 10–15% and this difference explains more than 90% of the difference in overall performance between male and female athletes (Laaksonen et al., 2018; Luchsinger et al., 2018, 2019). In the present study, despite the greater relative weight of the rifle for the females compared to the males (5.6 vs. 5.0% of body weight), no sex differences in absolute or relative responses to rifle carriage were identified. This is inconsistent with previous research showing that female biathletes had greater differences than males in $\dot{V}$_E_ (Stöggl et al., 2015) and $\dot{V}$O_2_ (Rundell and Szmedra, 1998) between WR and NR. This difference in findings may be explained by the greater difference in body mass between the male and female biathletes in the study by Rundell and Szmedra (1998) compared to the present study (∼ 16 kg vs. ∼ 7 kg).

## Conclusion

As hypothesized, rifle carriage increased the respiratory responses during submaximal roller-skiing, without any differences during maximal exercise. However, in contrast to our hypothesis, no sex differences were observed. In general, rifle carriage led to a decrease in the skiing speed at a blood lactate concentration of 4 mmol.L^–1^, as well as decreases in the anaerobic energy contribution and performance during a maximal TT lasting ∼ 3–4 min. The most important variables for predicting skiing speed in biathlon identified in the present study were the speed at 4 mmol.L^–1^ of blood lactate concentration and the anaerobic metabolic rate, both of which were lower when skiing with the rifle compared to without. Thus, to improve skiing performance in biathlon, improving skiing speed at 4 mmol.L^–1^ of blood lactate concentration and anaerobic energy delivery while carrying the rifle are recommended.

## Data Availability Statement

The datasets generated for this study are available on request to the corresponding author.

## Ethics Statement

The studies involving human participants were reviewed and approved by the Regional Ethical Review Board in Umeå, Sweden. The participants provided their written informed consent to participate in this study.

## Author Contributions

MJ initiated the study, collected and analyzed the data, and wrote the first draft of the manuscript. KM and ML contributed to the study design, data analysis, and writing of the manuscript. MJ, KM, and ML approved the final version to be published and agreed to be accountable for all aspects of the work.

## Conflict of Interest

The authors declare that the research was conducted in the absence of any commercial or financial relationships that could be construed as a potential conflict of interest.
